# Electrocardiogram Lead Placement Accuracy and Its Implications on Universal Screening in Athletes

**DOI:** 10.7759/cureus.88076

**Published:** 2025-07-16

**Authors:** Blake Boggess, Kari Kindschi, Eric Friedman, Samuel Boggess, David Berkoff

**Affiliations:** 1 Department of Orthopedics, Division of Sports Medicine, Duke University, Durham, USA; 2 Department of Sports Medicine, MedStar Health, Baltimore, USA; 3 Department of Sports Medicine, Charleston Sports Medicine and Orthopedic Center, Charleston, USA; 4 Department of Physical Medicine and Rehabilitation, University Hospitals, Cleveland, USA; 5 Department of Orthopedic Surgery, University of North Carolina at Chapel Hill School of Medicine, Chapel Hill, USA

**Keywords:** athlete, athletic heart syndrome, athletic preparticipation screening, cardiac ultrasound, electrocardiography (ecg)

## Abstract

Introduction: Abnormalities on the electrocardiogram (ECG) of athletes are common and are thought to reflect remodeling of the heart as an adaptation to physical training. Significant inter-individual variability exists in ECGs due to geometric factors-heart position, orientation, body habitus, height, and weight. Standard bony landmarks used for lead placement reflect chest wall anatomy, but not necessarily the true cardiac silhouette.

Methods: Forty male National Collegiate Athletic Association (NCAA) Division I athletes of varying body morphologies were enrolled in a prospective study. Eight different measurements were taken to define individual chest dimensions. An ECG was performed using standard lead placement based on bony landmarks. Cardiac ultrasound identified the left parasternal long view and the apical four-chamber view to represent a cardiac-based V2 and V4, respectively. The rest of the precordial leads were placed relative to these cardiac landmarks, and a second ECG was performed.

Results: The average distance between bony and cardiac-based leads was 29.8 mm (V2) and 58 mm (V4). For most subjects, cardiac V2 moved superiorly. With the exception of five subjects, cardiac V4 moved medially and superiorly. Wilcoxon's signed-rank test was performed for the P, QRS, and T wave axes as well as the amplitude of the R, S, and T waves, ST segment, and R:S ratio for the precordial leads. Cohen's d was used to determine the effect size. There was a consistent and significant difference between bony and cardiac ECGs in the R, S, T, and ST segment amplitudes in leads V2, V3, and V4. Despite the significant movement of precordial leads and amplitude changes, the ECGs based on the cardiac silhouette did not reveal any new abnormalities or changes in the abnormalities already present on the conventional ECGs.

Conclusion: There is a statistically significant difference in ECG lead measurements from the cardiac silhouette when compared with the standard bony landmarks. However, the overall interpretation of ECGs did not suggest any clinically significant alterations when leads are shifted to correlate with cardiac anatomy. Therefore, the study does not support the clinical use of cardiac silhouette over standard bony landmarks for ECG lead placement for athletic screenings or otherwise.

## Introduction

Sudden cardiac death (SCD) in a young, presumably healthy athlete can have profound impacts on individual families as well as various social and sports communities. Highly publicized events have focused attention on the challenges of pre-participation screening. Most of the cardiovascular conditions responsible for sudden cardiac death are asymptomatic [1]. SCD is the presenting symptom in many cases [2,3]. Exercise appears to be a trigger for lethal ventricular arrhythmias in predisposed athletes [4].

The appropriate screening method to identify athletes at risk for life-threatening cardiovascular events is controversial. The International Olympic Committee recommends a 12-lead electrocardiogram (ECG) prior to Olympic sport competition [5]. Major League Baseball, the National Hockey League, and the National Football League commonly use the ECG as part of pre-participation physicals [5]. In Italy, competitive athletes are screened regularly with a history, physical examination, and an ECG performed and interpreted by physicians with specialized training [4]. Since the Italian protocol was established, SCD due to cardiomyopathies has decreased by almost 90% [5-7]. Based on the Italian experience, the European Society of Cardiology recommends the implementation of a common screening protocol that features the 12-lead ECG [4]. Although the Italian results demonstrate significant benefit, there has been intense debate about the ability to translate the success to pre-participation screening in the United States. The American Heart Association does not advise screening ECGs, citing financial concerns, an insufficient framework for comprehensive evaluation of millions of athletes across the nation, and the lack of normative data for women and minorities. The association also notes the high frequency of false positives triggering additional tests, procedures, and anxiety [8].

Abnormalities on the ECGs of athletes are common and reflect the structural and electrical remodeling of the heart as an adaptation to physical training [1,2,6,7,9-13]. It can be difficult to differentiate between benign physiologic changes associated with Athletic Heart Syndrome and pathologic abnormalities of underlying cardiovascular disease [1]. ECGs of competitive athletes commonly display alterations in rhythm, conduction, repolarization, and precordial voltages [1-3,6,7,9,10,12,14-16]. The frequency of these variations depends on the type of training as well as the athlete's gender and race [6,11,12]. In a large study of 1005 Italian athletes across 38 sports, ECGs were compared to cardiac morphology evaluated by echocardiography. A spectrum of abnormal ECG patterns was present in 40% of the athletes, but structural disease was rarely responsible for the ECG alterations [12]. Most of the changes were due to the innocent consequences of athletic heart syndrome [12]. Male athletes and those involved in endurance sports have consistently demonstrated a greater number of ECG abnormalities [1,2,6,12,13]. In a study of almost 2000 football players participating in the NFL Invitational Camp, African American athletes demonstrated abnormal ECG patterns more often than their Caucasian counterparts [11].

Geometric factors, including heart position, orientation, body habitus, height, and weight, are also thought to contribute to the inter-individual variability of ECGs [17]. Cardiac MRI has been used to better understand the influence of these factors on each other and the electrical axis of the ECG. One study demonstrated significant variability in the heart’s position relative to lead V2 [18]. Others have found correlations between BMI and the orientation of the heart [19,20]. A strong relationship between the anatomic axis of the heart and the electrical axis of the ECG, however, has not been easy to define [19].

The aim of this study was to compare ECGs based on chest wall landmarks with ECGs using the cardiac silhouette as a guide for lead position. If individualized electrode placement with cardiac silhouette guidance significantly changes the ECG lead placement in athletes, it could provide further support for the inclusion of electrocardiograms in pre-participation screening protocols. The hypothesis is that if ECG leads were placed based on the cardiac silhouette, large variability would be seen in lead placement as compared to using standard bony landmarks. We then aim to evaluate if any variability seen suggests clinical significance in utilizing cardiac silhouette guidance in ECG lead position.

This article was previously presented as a meeting abstract at the 2010 AMSSM Annual Meeting on April 18, 2010.

## Materials and methods

The study protocol was approved by the Duke University Health System Institutional Review Board. A total of 40 volunteers were enrolled into this prospective study after providing written informed consent. The subjects were National Collegiate Athletic Association (NCAA) Division I male athletes without known heart disease. The inclusion criteria were male athletes at Duke University, ages 18-25, and height between five and seven feet. The exclusion criteria were any known prior cardiac disease. The data were collected from 12/3/2009 to 3/9/2010 at the Duke University Hospital emergency department. 

Data collection

The subject's height and weight were recorded, and body mass index was calculated. While supine on the exam table, the following measurements were taken in cm to define chest dimensions: sternal length (sternal notch to xiphoid process), sternal notch to left acromioclavicular (AC) joint, xiphoid to left AC joint, AC joint to AC joint, left AC joint to left inferior rib margin at the axillary line, left anterior axilla to iliac crest at the axillary line, chest circumference at xiphoid process, and chest circumference at inferior rib margin. 

The subjects had two ECGs performed; one based on standard bony landmarks and one based on the cardiac silhouette as identified on cardiac echocardiogram. Both sets of ECG leads were placed by the same investigator. In order to confirm the correct location of the electrodes for the bony landmark ECG, a linear GE LOGIQ e ultrasound probe (GE HealthCare, Chicago, IL) was used to identify the fourth intercostal space for V2. ECG electrodes were then placed relative to V2 in the conventional lead placement (V1: fourth intercostal space to the right of the sternum, V2: fourth intercostal space to the left of the sternum, V3: directly between V2 and V4, V4: fifth intercostal space at the midclavicular line, V5: level with V4 at the left axillary line, V6: level with V5 at the left midaxillary line). The positions of V2 and V4 were marked on the skin. Once all leads were placed, an ECG based on bony landmarks was performed. With the subject still supine, a cardiac GE LOGIQ e ultrasound probe was used to locate the cardiac silhouette and specifically, the left parasternal long view and the apical four-chamber view. Echocardiography was performed in the supine position, which differs from the standard position (left lateral decubitus). The purpose was to identify the cardiac silhouette in the supine position, the standard position for ECG lead interpretation. The ultrasound images were done by a qualified physician with ultrasound fellowship training. These views corresponded to a cardiac V2 and cardiac V4, respectively. The positions of these cardiac leads were marked on the skin. The distance between the bony V2 and cardiac V2 was measured, and the direction of movement was recorded. This was repeated for the bony V4 and cardiac V4. After these measurements, ECG leads V1, V3, V5, and V6 were placed relative to cardiac V2 and cardiac V4 with the same spatial relationship as in a conventional ECG for a second, cardiac landmark-based ECG.

Data analysis

Each 12-lead ECG was performed on a Philips PageWriter Touch (Philips HealthCare, Amsterdam, Netherlands) and recorded at 25 mm/s in the supine position during quiet respiration. The ECGs were analyzed for ventricular rate, interval measurements (PR interval, QRS duration, and QTc interval), and axis calculations (P, QRS, and T wave axes) using the Philips 12-Lead Algorithm. The amplitudes of precordial R, S, and T waves were measured individually. Precordial ST-segment elevation was measured at the J point on each ECG. The pattern and morphology of precordial waves were compared between the bony and cardiac landmark ECGs for each subject by a cardiologist blinded to identity, race, and sport. Additionally, the cardiologist was blinded to the fact that ECGs were bony landmark vs cardiac silhouette measurements.

In addition to standard descriptive statistics, statistical data analysis was performed. Means and standard deviations were calculated for the data points collected from both participant anatomical measurements and ECG values. Wilcoxon's signed-rank tests were then used to determine statistical significance for the measurements. Wilcoxon's signed-rank tests were used to identify p-values because the test can analyze the differences in non-normally distributed data, as was seen in this study's data. The ECG values were then analyzed using Cohen's d to determine the effect size of the differences. Cohen's d values of less than 0.2 were listed as having no effect, 0.2 through <0.5 as a small effect, 0.5 through <0.8 as a medium effect, and 0.8 and above as a large effect.

## Results

Demographic data of the study participants are outlined in Table 1. The study population included 20 Caucasian athletes, 19 African American athletes, and one Latino American athlete. Seven subjects played lacrosse, 32 played football, and one was a fencer. The age of the participants ranged from 18 to 23 years old.

**Table 1:** Demographic data of study participants.

Self-identified race	Number of participants
Caucasian	20 (50%)
African American	19 (45%)
Latino American	1 (5%)
Sport	
Football	32 (80%)
Lacrosse	7 (17.5%)
Fencing	1 (2.5%)
Age range	
18-23	40 (100%)

Values for the participants' heights, weights, and BMI were recorded. The averages, standard deviations, and ranges are shown in Table 2.

**Table 2:** Participant height, weight, and BMI. *Indicates not normally distributed.

Measurement	Mean ± standard deviation	Range
Height (cm)	188.08 ± 7.04	172.7–200.7
Weight (kg)	105.55 ± 20.87*	70.3–143.8
BMI (kg/m^2^)	29.6 ± 4.30*	22.8–40.2

Values for the various participant chest dimensions are seen in Table 3.

**Table 3:** Participant chest dimensions. *Indicates not normally distributed. AC: acromioclavicular.

Anatomical distance measured	Mean ± standard deviation	Range
Sternal notch to xiphoid process (cm)	21.68 ± 2.44	17.0–26.5
Sternal notch to left AC joint (cm)	21.61 ± 1.96*	19.0–25.5
Xiphoid to left AC joint (cm)	34.64 ± 2.64	28.5–40.0
AC joint to AC joint (cm)	39.76 ± 3.30	33.5–46.5
Left AC joint to left inferior rib margin at the axillary line (cm)	38.48 ± 3.09	34.0–47.0
Left anterior axilla to iliac crest at the axillary line (cm)	38.81 ± 2.67	30.0–44.0
Chest circumference at the xiphoid process (cm)	98.76 ± 10.44*	82–120
Chest circumference at the inferior rib margin (cm)	93.51 ± 11.75*	77.5–118

The differences between the ECG lead placement from bony landmarks and the ultrasound cardiac silhouette were marked and recorded. The results for the two leads placed with ultrasound-guidance (V2 and V4) are seen in Table 4.

**Table 4:** Distance of lead placement differences.

Leads measured	Mean ± standard deviation	Range
Distance of V2 (mm)	29.82 ± 17.97	2–64
Distance of V4 (mm)	58.02 ± 24.18	3–127

The superior/inferior directional changes from bony landmark to cardiac silhouette placement are seen in Table 5.

**Table 5:** Anatomical superior/inferior directional change in ECG leads.

Directional change	Superior	Inferior	No change
V2	34 (85%)	2 (5%)	4 (10%)
V4	36 (90%)	0 (0%)	4 (10%)

The medial/lateral directional changes from bony landmark to cardiac silhouette placement are seen in Table 6.

**Table 6:** Anatomical medial/lateral directional change in ECG leads.

Directional change	Medial	Lateral	No change
V2	19 (47.5%)	9 (22.5%)	12 (30%)
V4	39 (97.5%)	1 (2.5%)	0 (0%)

The ECG data are represented in Table 7 and include both the mean and standard deviation for the bony and cardiac ECG measurements. The Wilcoxon signed-rank test determined the p-value, and a p-value <0.05 was considered significant. The strength of the difference and the effect size were derived from Cohen's d. The most notable trend was seen in leads V2-V4. Except for the V2 S wave and the V4 T wave, the amplitudes of R, S, T, and ST segments were significant across leads V2, V3, and V4. The effect size was varied across the dataset. Notably, there were only two large effect size differences, seen in V4 R amplitude and V4 S amplitude. Looking at each wave collectively across all precordial leads, the difference between the bony and cardiac ECGs was significant in the R and S waves. The corresponding effect size was medium for the sum amplitude of the R waves and small for the sum amplitude of the S wave.

**Table 7:** ECG value comparison of bony landmark placement vs cardiac silhouette placement. *Indicates not normally distributed. P-value tested using Wilcoxon's signed-rank test. amp: amplitude, seg amp: segment amplitude.

ECG value	Bony ECG: Mean ± standard deviation	Cardiac ECG: Mean ± standard deviation	p-value	Cohen's d	Effect size
Heart rate (bpm)	70.6 ± 12.74	69.02 ± 12.36	0.032	0.1259	None
PR interval (msec)	171.4 ± 21.54	173.3 ± 22.40	0.077	0.0865	None
QRS interval (msec)	96.35 ± 12.12*	97.25 ± 11.98*	0.061	0.0747	None
QTc (msec)	419.32 ± 21.52	418.68 ± 20.38	0.926	0.0305	None
P axis (deg)	46.6 ± 25.68*	43.3 ± 28.03	0.056	0.1228	None
QRS axis (deg)	59.12 ± 24.81	60.38 ± 26.59	0.202	0.0490	None
T axis (deg)	30.62 ± 15.33	33.32 ± 22.92*	0.834	0.1385	None
V1 R amp (mV)	0.45 ± 0.33*	0.44 ± 0.29*	0.497	0.0322	None
V2 R amp (mV)	0.94 ± 0.45*	0.89 ± 0.45*	0.027	0.1111	None
V3 R amp (mV)	1.2 ± 0.55*	1.02 ± 0.44*	0.009	0.3614	Small
V4 R amp (mV)	2.02 ± 0.56*	1.27 ± 0.58	<0.001	1.3156	Large
V5 R amp (mV)	1.9 ± 0.50*	1.82 ± 0.44	0.068	0.1699	None
V6 R amp (mV)	1.44 ± 0.34	1.43 ± 0.31*	0.659	0.0307	None
V1 S amp (mV)	1.32 ± 0.64*	1.37 ± 0.57*	0.276	0.0825	None
V2 S amp (mV)	2.04 ± 0.89	2.00 ± 0.86	0.296	0.0457	None
V3 S amp (mV)	1.32 ± 0.87*	1.75 ± 0.93*	<0.001	0.4775	Small
V4 S amp (mV)	0.77 ± 0.48	1.34 ± 0.79*	<0.001	0.8720	Large
V5 S amp (mV)	0.25 ± 0.20*	0.29 ± 0.21*	0.062	0.1951	None
V6 S amp (mV)	0.11 ± 0.11*	0.12 ± 0.11*	0.156	0.0909	None
V1 T amp (mV)	0.07 ± 0.19*	0.05 ± 0.19*	0.081	0.1053	None
V2 T amp (mV)	0.67 ± 0.29	0.61 ± 0.32	<0.001	0.0655	None
V3 T amp (mV)	0.70 ± 0.31	0.69 ± 0.31	<0.001	0.0323	None
V4 T amp (mV)	0.63 ± 0.30*	0.68 ± 0.31	0.054	0.1639	None
V5 T amp (mV)	0.46 ± 0.17	0.50 ± 0.19*	0.016	0.2219	Small
V6 T amp (mV)	0.34 ± 0.11*	0.36 ± 0.13*	0.075	0.1661	None
V1 ST seg amp (mV)	0.066 ± 0.041*	0.064 ± 0.044*	0.825	0.0470	None
V2 ST seg amp (mV)	0.174 ± 0.066*	0.160 ± 0.067*	0.026	0.2105	Small
V3 ST seg amp (mV)	0.145 ± 0.070*	0.178 ± 0.070*	0.001	0.4714	Small
V4 ST seg amp (mV)	0.105 ± 0.058*	0.148 ± 0.062*	<0.001	0.7163	Medium
V5 ST seg amp (mV)	0.079 ± 0.104*	0.084 ± 0.074*	0.148	0.0554	None
V6 ST seg amp (mV)	0.035 ± 0.028*	0.065 ± 0.103*	0.060	0.3975	Small
Sum R amp V1-V6 (mV)	7.96 ± 1.97	6.86 ± 1.86	<0.001	0.5742	Medium
Sum S amp V1-V6 (mV)	5.81 ± 2.36	6.87 ± 2.78*	<0.001	0.4111	Small
Sum T amp V1-V6 (mV)	2.88 ± 1.13	2.89 ± 1.23	0.628	0.0085	None

From the cardiology ECG review, the overall morphology of the tracings did not change when the bony and cardiac ECGs were compared for each athlete. The cardiac landmark-based ECG did not normalize or significantly alter any of the abnormalities present on the bony ECGs. 

## Discussion

Conventional placement of the ECG leads is based on intercostal spaces and chest wall anatomy. Bony landmarks are thought to represent cardiac landmarks, but confirmatory studies are lacking. This study examines the relationship between the standard leads and the cardiac silhouette. The results from the study support the hypothesis that large variability would be seen in lead placement when using the cardiac silhouette compared to standard bony landmarks. The significant movement of the cardiac-based V2 and V4, averaging almost 3 and 6 cm, respectively, indicates that traditional bony landmarks do not accurately reflect the heart’s position in many athletes. The results suggest that V4, in particular, could be quite sensitive to anatomical variations. Though some of the individual measurements were statistically significant (such as V4 R and S amplitudes), the overall values of the Cohen's d effect size and lack of overall ECG morphology changes suggest a lack of clinical significance.

The ECGs of athletes demonstrate significant inter-individual variability for many reasons. Physiological remodeling, as well as geometric factors (heart position, orientation, body habitus), gender, and race, are all thought to contribute. Tailoring the electrodes to the individual's cardiac silhouette resulted in significant differences in lead amplitude but did not change the morphology of the ECG. In almost every instance, the V4 lead moved medially and superiorly. Not surprisingly, its pattern in the cardiac ECG was similar to the bony V3 pattern. Overall, the cardiac-based ECGs did not demonstrate any new abnormalities or any significant decrease in variability with the individualized electrode placement. These findings support earlier studies using cardiac MRI to position a grid of electrodes relative to heart position in the frontal plane [18].

The high frequency of ECG alterations associated with Athletic Heart Syndrome affects the sensitivity and specificity of the screening ECG in athletic populations [8]. The consequences of both under- and over-interpretation of ECGs are significant [5]. Despite attempts to define the limits of normal among athletes [9], false positive results continue to represent a limitation to the use of ECGs as a mass-screening tool. Per the cardiology evaluation in this study, there were no noted changes in common false-positive patterns, such as early repolarization or voltage criteria for left ventricular hypertrophy. The use of ultrasound to personalize lead placement increases the costs and time associated with screening without improving ECG accuracy. The lack of readily available ultrasound equipment and physicians trained to interpret the results makes its use for this purpose impractical.

There are several limitations to the study. An athlete's ECG is the result of a complex and distinctly individual interaction between both physiologic and geometric factors. It is difficult for ultrasound to account for the heart’s orientation and rotation around the anatomic axis. Ultrasound also does not evaluate the Purkinje system or heart muscle fiber orientation, both of which may contribute to inter-individual variability [17,19]. Our reference images for cardiac V2 and V4 come from traditional echocardiogram views. The left parasternal long view is usually performed with the patient in the left lateral decubitus position. The cardiac ultrasound in this study was always performed with the subject supine to reflect the usual positioning for ECGs. Another limitation was the small number of participants (40 total) in the study. Additionally, the study did not include female athletes and had a limited number of endurance athletes. While there was a broad range of body shapes and sizes, our mean BMI was 29.6, which reflected that the majority of our athletes were football players. Another limitation is the skewed participation in football compared to other sports.

Future studies may focus on chest dimensions and investigate the possibility of a correction factor for body morphology that could be used to reduce the influence of geometric factors on the inter-individual variability of ECGs. For example, utilizing a more narrow range for participant BMI or evaluating for any relationship between BMI and lead placement. Other examples include investigating potential differences in ECG variability in racial groups, athlete sex, or athlete sport. Ultrasound could also be utilized to identify the anatomy behind each electrode to further document the relationship between the bony landmarks and the cardiac silhouette in individuals. Lastly, a future consideration is studying variability in ECG lead placement in the broader non-athlete population.

## Conclusions

In review, the study found that the cardiac silhouette does have statistically significant differences in ECG values when compared with the standard bony landmarks used for ECG placement. Though statistically significant, the effect sizes seen from the Cohen's d values suggest the ECG differences are unlikely to be clinically significant. When the leads were shifted to correlate with cardiac anatomy in the study, the overall morphology of the ECG was not significantly altered in any of the 40 male participants in the study. While this study does not answer the debate regarding the use of pre-participation ECG screening for athletes, the study does not support the use of ultrasound to personalize ECG lead placement based on cardiac silhouettes. This stems from the lack of significant alterations in the ECG morphologies and the impractical additional cost with the ultrasound use.
